# Association between Work Related Stress and Health Related Quality of Life: The Impact of Socio-Demographic Variables. A Cross Sectional Study in a Region of Central Italy

**DOI:** 10.3390/ijerph15010159

**Published:** 2018-01-19

**Authors:** Giuseppe La Torre, Cristina Sestili, Alice Mannocci, Alessandra Sinopoli, Massimiliano De Paolis, Sara De Francesco, Laura Rapaccini, Marco Barone, Valentina Iodice, Bruno Lojodice, Sabina Sernia, Simone De Sio, Angela Del Cimmuto, Maria De Giusti

**Affiliations:** 1 Department of Public Health and Infectious Diseases, Sapienza University of Rome, 00185 Rome, Italy; cristina.sestili@uniroma1.it (C.S.); alice.mannocci@uniroma1.it (A.M.); alessandra.sinopoli@uniroma1.it (A.S.); massi.7288@hotmail.it (M.D.P.); saradf22@gmail.com (S.D.F.); raplaura@libero.it (L.R.); marcobarone92@gmail.com (M.B.); valentina.iodice.1992@hotmail.it (V.I.); bruno.lojodice@uniroma1.it (B.L.); sabina.sernia@uniroma1.it (S.S.); angela.delcimmuto@uniroma1.it (A.D.C.); maria.degiusti@uniroma1.it (M.D.G.); 2 Research Unit of Occupational Medicine, Sapienza University of Rome, 00185 Rome, Italy; simone.desio@uniroma1.it

**Keywords:** quality of life, Italy, job demands-control model, occupational stress, workers

## Abstract

The aim of this work is investigate relationship between health-related quality of life and work-related stress and the impact of gender, education level, and age on this relationship. A cross-sectional study was conducted among workers of various setting in Rome and Frosinone. Work-related stress was measured with a demand–control questionnaire and health-related functioning by SF (short form)-12 health survey. There were 611 participants. Men reported high mental composite summary (MCS) and physical composite summary (PCS). In multivariate analysis age, gender (*p* < 0.001) and job demand (0.045) predicted low PCS. Low MCS predicted poor PCS. Job demand and educational level resulted negatively associated with MCS. In an analysis stratified for age, gender, and educational level, gender and age resulted effect modifier for MCS, gender and education level for PCS. In women increase of decision latitude predict (*p* = 0.001) an increase in MCS; a low job demand predict high MCS in male (*p* ≤ 0.001). In younger workers, a lower level of job demand predicted high MCS (<0.001). For PCS, gender and education level resulted effect modifier. In women, high decision latitude predicted higher PCS (*p* = 0.001) and lower level of job demand results in higher PCS (*p* ≤ 0.001). Higher educational level resulted predictor of low PCS. Management of risk about work-related stress should consider socio-demographic factors.

## 1. Introduction

Work-related stress is a serious occupational health problem and has been subject to a large amount of research and interest in recent years [1,2,3]. Workplace stress is the physical and emotional response that occurs when job demands are in conflict with the ability, resources or needs of the worker [4]. People spend much of their time at work, and the work environment has a strong impact on psycho-physiological and social wellbeing. Furthermore, work-related stress could have a negative impact on the health of employees and work organizations [5]. Moreover, stress at work is one of the major costs to companies and countries, deeply affecting productivity. Work related stress can hit individuals in various way and is important to consider sociodemographic factors approaching stress-related issues. According to the Sixth European Working Conditions Survey carried out in 2015, structural inequalities and differences in terms of gender, employment status, and occupation were significant [6]. In recent years, some studies investigated possible indicators of the social and occupational determinants of health [7,8,9]. To our knowledge, only a few studies investigated whether physical composite summary (PCS) and mental composite summary (MCS) are modified by age, gender, and educational level. The aim of this study was to determine whether or not there is a relationship between health-related quality of life and work related stress and to explore if socio-demographic characteristics have an influence on this relationship. These hypotheses were tested in a population of workers of some cities in the Lazio region (Italy). 

## 2. Methods

### 2.1. Study Design

A cross-sectional study was carried from July 2014 to June 2015, following the STROBE Statement [10].

### 2.2. Setting 

Provinces of Frosinone and Rome, Lazio Italy.

### 2.3. Participants

The study was conducted among various types of workers of some companies (teachers, employees in a supermarket, white collar workers, blue collar workers, and health personnel) administering a structured questionnaire. Participation was voluntary and anonymous. Individuals were eligible to take part to study if they were between 18–65 years old and if they were workers in described setting. Questionnaires were left in each workplace for one week. The subjects resided in the region of Lazio (provinces of Rome and Frosinone). All subjects gave their informed consent for inclusion before they participated in the study. The study was conducted in accordance with the Declaration of Helsinki, and the protocol was approved by the Ethics Committee of Sapienza University of Rome (Project identification code 4268). The participants were given a period of seven days to fill in the questionnaire and a closed box was left at the workplace so that respondents could place their filled questionnaires anonymously. After that period, a collaborator returned to collect the questionnaires.

### 2.4. Questionnaires

The validated questionnaire was composed from various sections. The first section was composed from socio demographic data, followed by the Short-Form 12 Health Survey, a short version of the SF-36 questionnaire [11,12]. The items of the SF-12 were selected to reproduce the two summary measures, physical component summary (PCS) and mental component summary (MCS), of the SF-36. It contains eight subscales as original 36-item questionnaire: physical functioning (PF, 2 items), role limitations due to physical problems (RP, 2 items), bodily pain (BP, 1 item), general health perceptions (GH, 1 item), vitality (VT, 1 item), social functioning (SF, 1 item), role limitations due to emotional problems (RE, 2 items) and mental health (MH, 2 items) [13]. PCS and MCS are computed using the scores of the 12 questions and range from 0 to 100, with 0 indicating the lowest level of health measured by the scales and 100 indicating the highest level of health-related quality of life.

Another part was composed from the Karasek Job Content Questionnaire (JCQ). JCQ is a standardized instrument proposed to measure the dimensions of the demand–control model [14]. It is widely used to evaluate psychosocial factors at work. The model is constructed on the relationship that exists between high work demand, defined job demand, and decision latitude (freedom of decision), which defines a job strain status (perceived work stress). The job content questionnaire (JCQ) included the following recommended format: 49 questions (scales of decision latitude—skill discretion and decision authority, psychological demand, physical demand, social support—supervisor and coworker support, and job insecurity). In order to build indicators, for each scale of the questionnaire, a sum of the weighted item score was calculated according to the user’s guide of the job content questionnaire. Job demand measured quantitative and qualitative workload. Decision latitude was done by combining the items from the skill discretion (three items) and decision authority (three items) scales. Using Cartesian coordinates, the results of the questionnaire system is used to divide the population into four quadrants: workers with high perceived stress (highest score in demand, low control); active (high demand, high control); passive (low demand, low control); and subject (high demand low control).

### 2.5. Statistical Analysis

Statistical analysis of the data was conducted using the statistical software SPSS, release 23.0 (IBM Corporation, Armonk, NY, USA). Univariate analysis included Student *t*-test for quantitative and χ^2^ test for qualitative variables. The independent variables used in this study were gender, age, educational level, type of work, and seniority job. Age was divided into three five-year groups for men and four five-year groups for women. Three categories were defined for educational level, ranging from junior high school to University degree. 

The dependent variables in this study were PCS and MCS. For the PCS, very low scores indicate substantial limitations in self-care, physical, social, and role activities; severe body pain; or frequent tiredness. For the MCS, very low scores indicate frequent psychological distress, and substantial social and role disability due to emotional problems. Further analyses were conducted to evaluate association between decision latitude, job demand, and two outcome variables. Multivariate analysis was conducted with a backward stepwise approach (dependent variable MCS and PCS). Goodness of fit of the models was evaluated with R^2^, Akaike information criteria, Bayesian Swatz criteria, and Amemiya prevision criterion. Finally, for assessing if an effect modification does exist, analysis was stratified for gender, educational level, and age. The statistical significance was set at *p* < 0.05 for all analyses. 

## 3. Results

Six-hundred and forty-nine workers were invited to participate and 611 entered the study (response rate 91.3%); 59.9% (366) were males and 40.1% (245) were females. Workers were 50 teachers (8.2%), 162 white collar workers (26.5%), 310 blue collar workers (50.7%), and 89 health professionals (14.6%). Table 1 describes the socio-demographic and occupational characteristics of the workers by gender. Most of the male workers were in the category of 35 to 44 years (32.6%) and 6.3% of the men were older than 55 years. Most of the women were in the 45–54 (31.4%) group of age and 7.9% were older than 55. As far as concerns educational level, 70.5% of the sample had a senior high school degree. Most of the male and female workers have more than 10 years of work experience.

**Figure 1:**
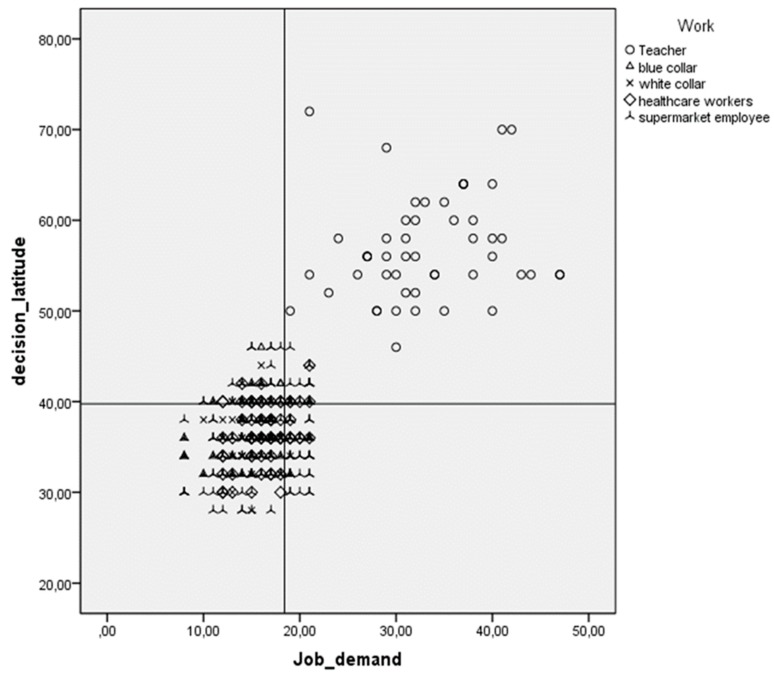
Study population split in four quadrants according to Karasek.

Scores of decision latitude and job demand using Cartesian coordinates were used to split the population into four quadrants (Figure 1). Workers at high stress risk (high strain) in quadrant IV were supermarket employees and healthcare workers.

Men reported lower educational level than women (*p* < 0.001). Women have more years of occupation than men (*p* < 0.001). Female workers have significantly higher decision latitude and job demand levels than the male workers (*p* < 0.001). Although Student *t*-test revealed no statistical difference between men and women in MCS and PCS, men reported a higher score. Means of MCS and PCS in men were, respectively, 48.18 and 51.1. In women, MCS and PCS were 47.6 and 50.3. The medians of the whole population were 52.85 for PCS and 50.07 for MCS. This result is over the mean of the general population (49.6 and 48.8 respectively) [15]. 

Univariate analysis shows no statistical significant differences between type of worker for PCS (*p* = 0.466) and MCS (*p* = 0.130) score. For decision latitude and job demand, univariate analysis shows significant differences (*p* ≤ 0.001) (data not shown). 

In order to test the (linear) main and interactive effects of the variable on PCS and MCS sets of hierarchical multiple regression analyses were performed separately for PCS and MCS (Table 2).

Age (*p* = 0.004, β = −0.124), gender (*p* < 0.045, β = −0.088), and job demand (*p* ≤ 0.001, β = −0.0340) are negatively associated with PCS. A high job demand score and being older predict a low score of PCS. Subjects with a low score of MCS were more likely to have poor PCS. Job demand (*p* ≤ 0.001, β = −0.306) and educational level (*p* = 0.001, β = −0.143) are negatively associated with MCS, i.e., high educational level and high job demand are predictor of low MCS. 

To verify if there is an effect modifier analysis was stratified for age (<45 years and >45 years), gender, and educational level (lower and higher) (Table 3). For MCS, gender and age result as effect modifiers. Among women, increasing decision latitude predicts (*p* = 0.001) an increase in MCS (not in man); a low job demand score predicts a high MCS in male workers (*p* ≤ 0.001). In younger (<45 years) workers lower level of job demand predicts high MCS (<0.001). For PCS, gender and education level act as effect modifiers. Among women high decision latitude predicts higher PCS (*p* = 0.001) and lower level of job demand results in high level of PCS (*p* ≤ 0.001). A higher educational level is a predictor of low PCS.

## 4. Discussion 

This study explored the relationship between socio-demographic characteristics, type of work, and perceived risk for work related stress measured using validated version of questionnaire (SF12 and JCQ). The findings confirmed that some socio-demographic characteristics are associated with work-related stress risk factors. This result is in line with exiting literature [16,17,18,19,20,21,22]. Moreover, the stratified analyses clearly show that gender, age, and educational level act as effect modifiers of the association between decision latitude/job demand and both PCS and MCS. Our analyses indicate that a high level of job demand is more harmful for physical wellbeing of women and high decision latitude increases physical and mental wellbeing in women. A study by Rivera Torres [23] found that the generation of job stress has a different pattern in men and women. Several studies documented that female workers suffer more stress than male workers and report lower health status than men [24]. Age is an important factor due to the increasing number of older workers, in relation to increasing life expectancy. In this work, age results effect MCS, probably due to older workers being expected to react in a different way from younger workers to work stress. In younger workers, high job demand is associated with lower MCS in a significant way. Both for younger and older workers, high job demand predicts a low PCS in a significant way [25,26]. Burr [27] found that work demands have a stronger impact on the health of older compared to younger workers and, in the relationship between physical work demands with health, age resulted as an effect modifier. According to an Italian law from 2008, there is obligation for companies to assess work related stress risk. A study proposed objective measurement tool such as HSE (Health and Safety Executive) indicator tool, for more appropriate prevention measures [28]. As for interventions aimed at reducing stress in health workers, the most recent review indicates that there is moderate evidence that changing work schedules, cognitive-behavioral training, and mental and physical relaxation reduce stress [29]. Literature indicates that health promotion interventions must be founded on evidence-based principles [30] and, if well-designed and well-executed, can achieve positive health and financial outcomes [31].

## 5. Conclusions

The results suggest the importance of some socio-demographic variables for work-related stress. This study shows that age, gender, and educational level influence the association between work-related stress and MCS/PCS. This result could be of great interest for the workplace health promoter who could prevent and manage work-related stress considering specific socio-demographic variables in the workers’ health surveillance.

### Study Limitations

Our study has several limitations. Firstly, the cross-sectional nature of study could not find a causal inference on causality or temporal ordering of variables. A longitudinal analysis is required. Secondly, both exposure to job stress and health-related quality of life were self-reported, and more objective measurements are needed in future studies. In fact, the main limitation of self-reported questionnaires is that they provide “subjective” measures, representing the occupational stress perceptions of individual workers. Another limitation is the lack of data on ethnicity of participants. 

## Figures and Tables

**Table 1:** Characteristics of the sample population by gender.

Variables	Males (Tot. *n* = 366)	Females (Tot. *n* = 245)	*p*
*Age groups*			
<25	30 (82%)	24 (9.8%)	0.431
25–34	90 (24.6%)	52 (21.2%)	
35–44	126 (34.4%)	73 (29.8%)	
45–54	97 (26.5%)	77 (31.4%)	
55–66	23 (6.3%)	19 (7.8%)	
MCS 12	48.18	47.6	0.695
PCS 12	51.1	50.3	0.953
Decision latitude	36.4	40.8	<0.001
Job demand	16.0	19.4	<0.001
*Educational level*			
Junior high school	73 (19.9%)	32 (13.1%)	<0.001
Senior high school	267 (73.0%)	164 (66.9%)	
University degree	26 (7.1 %)	49 (20.0%)	
*Years of occupation*			
Up to 3 years	109 (29.8%)	44 (18.0%)	0.004
4–9 years	95 (26.0%)	78 (31.8%)	
10 years and over	162 (44.3%)	123 (50.2%)	

**Table 2:** Predictors of MCS 12 and PCS 12 in the whole study population.

Variables	Dependent Variable: MCS 12	Variables	Dependent Variable: PCS 12
	*Beta (p)*		*Beta (p)*
Age	0.009 (0.838)	Age	−0.124 (0.004)
Gender	−0.036 (0.404)	Gender	−0.088 (0.045)
PCS 12	0.302 (<0.001)	MCS 12	0.290 (<0.001)
Decision latitude	0.070 (0.367)	Decision latitude	0.097 (0.209)
Job demand	−0.306 (<0.001)	Job demand	−0.340 (<0.001)
Educational level	−0.143 (0.001)	Educational level	0.050 (0.254)
Teachers	0.381 (<0.001)	Teachers	0.293 (<0.001)
Tertiary workers	−0.016 (0.699)	Tertiary workers	0.065 (0.116)
*R^2^ of the model*	0.162	*R^2^ of the model*	0.165
*Akaike information criteria*	2321.829	*Akaike information criteria*	1940.2
*Amemiya prevision criterion*	0.854	*Amemiya prevision criterion*	0.853
*Bayesian Swartz Criterion*	2343.2	*Bayesian Swartz Criterion*	1970.2

**Table 3:** Predictors of MCS 12 and PCS 12 stratified by gender, age and educational level.

**Dependent Variable: MCS 12**	**Variables**	**Gender *Beta (p)***		**Age *Beta (p)***		**Education *Beta (p)***	
		**Males**	**Females**	**<45 Years**	**≥45 Years**	**Lower**	**Higher**
	PCS 12	0.277 (<0.001)	0.410 (<0.001)	0.223 (<0.001)	0.467 (<0.001)	0.434 (<0.001)	0.258 (<0.001)
	Decision latitude	−0.045 (0.421)	0.146 (0.031)	−0.104 (0.140)	0.123 (0.416)	0.138 (0.136)	−0.058 (0.531)
	Job demand	−0.145 (0.007)	−0.126 (0.284)	−0.264 (<0.001)	−0.159 (0.289)	−0.200 (0.025)	−0.304 (0.001)
	*R^2^ of the model*	0.12	0.177	0.14	0.234	0.334	0.122
	*Akaike information criteria*	1403.4	935.7	1532.4	786.1	418.03	1905.5
	*Amemiya prevision criterion*	0.902	0.855	0.881	0.791	0.722	0.894
	*Bayesian Swartz Criterion*	1418.540	949.2	1551.7	795.7	428.4	1921.8
**Dependent Variable: PCS 12**	**Variables**	**Gender *Beta (p)***		**Age *Beta (p)***		**Education *Beta (p)***	
		**Males**	**Females**	**<45 Years**	**≥45 Years**	**Lower**	**Higher**
	MCS 12	0.298 (<0.001)	0.338 (<0.001)	0.226 (<0.001)	0.409 (<0.001)	0.493 (<0.001)	0.253 (<0.001)
	Decision latitude	0.006 (0.977)	0.382 (0.001)	0.009 (0.169)	−0.006 (0.966)	−0.068 (0.443)	0.138 (0.136)
	Job demand	−0.066 (0.229)	−0.437 (<0.001)	−0.144 (0.023)	−0.496 (<0.001)	−0.139 (0.124)	−0.343 (<0.001)
	*R^2^ of the model*	0.08	0.29	0.11	0.29	0.29	0.132
	*Akaike information criteria*	1177.615	754.3	1278.4	649.9	349.8	1594.5
	*Amemiya prevision criterion*	0.923	0.744	0.916	0.748	0.746	0.888
	*Bayesian Swartz Criterion*	1185.1	774.5	1297.7	665.931	357.6	1614.9
